# A Low-Power RRAM Memory Block for Embedded, Multi-Level Weight and Bias Storage in Artificial Neural Networks

**DOI:** 10.3390/mi12111277

**Published:** 2021-10-20

**Authors:** Stefan Pechmann, Timo Mai, Julian Potschka, Daniel Reiser, Peter Reichel, Marco Breiling, Marc Reichenbach, Amelie Hagelauer

**Affiliations:** 1 Chair of Communications Electronics of University of Bayreuth, 95447 Bayreuth, Germany; 2 Institute for Electronics Engineering, Friedrich-Alexander University Erlangen-Nuernberg, 91058 Erlangen, Germany; timo.mai@fau.de (T.M.); julian.potschka@fau.de (J.P.); 3 Chair of Computer Science 3 (Computer Architecture), Friedrich-Alexander University Erlangen-Nuernberg, 91058 Erlangen, Germany; daniel.g.reiser@fau.de; 4 Fraunhofer Institute for Integrated Circuits (IIS), Division Engineering of Adaptive Systems EAS, 01187 Dresden, Germany; peter.reichel@eas.iis.fraunhofer.de; 5 Fraunhofer Institute for Integrated Circuits (IIS), 91058 Erlangen, Germany; marco.breiling@iis.fraunhofer.de; 6 Chair of Computer Engineering, Brandenburg University of Technology (B-TU), 03046 Cottbus, Germany; marc.reichenbach@b-tu.de; 7 Fraunhofer Institute for Microsystems and Solid State Technologies (EMFT), 80686 Munich, Germany; amelie.hagelauer@emft.fraunhofer.de; 8 Chair of Micro- and Nanosystems Technology, Technical University of Munich, 80333 Munich, Germany

**Keywords:** ANN, low-power, embedded memory, memory block, multi-level, RRAM

## Abstract

Pattern recognition as a computing task is very well suited for machine learning algorithms utilizing artificial neural networks (ANNs). Computing systems using ANNs usually require some sort of data storage to store the weights and bias values for the processing elements of the individual neurons. This paper introduces a memory block using resistive memory cells (RRAM) to realize this weight and bias storage in an embedded and distributed way while also offering programming and multi-level ability. By implementing power gating, overall power consumption is decreased significantly without data loss by taking advantage of the non-volatility of the RRAM technology. Due to the versatility of the peripheral circuitry, the presented memory concept can be adapted to different applications and RRAM technologies.

## 1. Introduction

According to the US Centers for Disease Control and Prevention, Atrial Fibrillation (AFib) is the most common type of treated heart arrhythmia [1]. It occurs when the heart chambers are not beating regularly and causes one in seven strokes. In an article published in 2017, it was even labeled as an epidemic that will be on the rise within the next 10 to 20 years due to the worldwide aging of the population, especially in Western countries, since AFib is more prevalent in older people. By 2060, it is predicted that up to 17.9 million people will be affected by AFib in Europe [2]. Therefore, the detection of AFib is a task with increasing importance. AFib can be detected by recognizing a specific pattern in the electrocardiogram (ECG) signal. A difficulty in detecting AFib lies in the property of some types of the affliction, that the symptoms occur in an irregular manner and may only happen during brief periods of time [3]. Therefore, monitoring the ECG signal by a wearable device and looking for the AFib pattern during a long period of time can help prevent major health problems in an increasing part of the population.

The usage of neural networks for detection of AFib patterns in ECG signals has been a common technique for some time, but research is still ongoing in this field [4,5]. ANNs are widely used in image and pattern recognition in general, since this type of computing approach is well suited for these tasks [6]. For detection of AFib, it is desirable to monitor the ECG signal of a person as long as possible while affecting the life of the patience as little as possible. So a wearable sensor device using ANNs for detecting ECG signal patterns can help these patients. To prolong the battery lifetime of such a device, low-power electronics are a necessity. Furthermore, switching off parts of the device during idle times can save power as well, since the ANN can be switched off, while sensor data are collected in the chip’s front-end and then switched on to process the acquired data in a batch. This is useful since the data processing can be done much faster than the data collection due to the relatively low bandwidth of the ECG signal compared to clock frequencies in electrical devices.

However, loading the necessary parameters of an ANN from an external memory takes a lot of energy, since the data have to be transferred between different ICs due to the fact that such parameters are usually stored in external memory. If the processing elements are switched off during idle times to save energy, this data transfer has to be performed at every power on, resulting in high energy consumption compared to the overall energy for the processing of the ANN. Therefore, the combination of embedded, non-volatile memory with neural networks has the potential to reduce the energy demands of suitable tasks such as image or pattern recognition significantly.

Resistive Random Access Memory (RRAM) is an emerging memory technology. It can offer non-volatility and energy efficiency during read operations [7]. When utilizing 3D integration, the cells could even be scaled to a chip area below $4F^{2}$ [8], where $F^{2}$ is the minimum feature size, which results in less chip area compared to other memory technologies such as SRAM. Furthermore, the multi-bit capability can increase the memory density. Resistive switching devices also gain attraction as an analog circuit element due to their hysteresis and found use in neuromorphic computing circuits [9]. By combining these three techniques—ANNs as energy-efficient computing method, switching off the system when idle, and using embedded RRAM memory for low-power parameter loading and flexibility due to programmability—it is possible to build efficient systems for data processing, especially in the field of wearable medical sensors for AFib detection.

**Figure 1:**
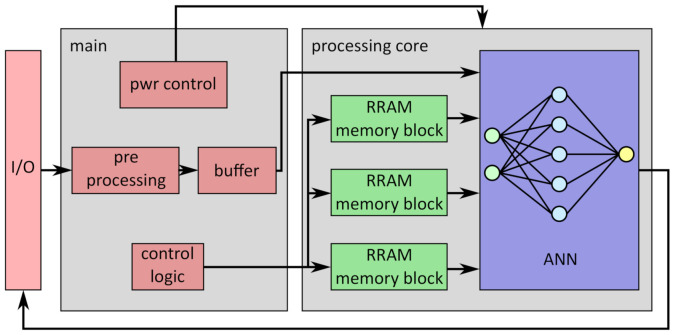
Overview of a system utilizing ANN (blue) and the presented RRAM memory block (green) for AFib detection in sampled ECG signals. The RRAM memory blocks store the weight and bias values for the ANN in a non-volatile way to enable power gating for the processing core in general and the memory blocks individually after loading the values into the ANN [12].

An example for the usage of the presented memory block is shown in Figure 1 [10,11]. The system contains two major parts:

- Main: contains the in- and output interface, deals with the preprocessing and buffering of the sampled ECG signal, and takes care of the power control and the logic to control the RRAM blocks;
- Processing core: contains the actual ANN for the inference of the data and several instances of the presented RRAM memory block for weight and bias storage.

The digital architecture consists of several hardware blocks, each of which represents a layer of the ANN. Each block uses simple processing elements that perform arithmetic operations (mainly multiplication and addition). The required parameters are directly routed in parallel from the RRAM memory blocks to the processing elements. Thus, all parameters need to be read in parallel. The processing core can be switched off by the main core in order to save energy. If enough input data are preprocessed and buffered, the processing core can be switched on; the weights and bias values stored in the RRAM blocks are then loaded and the buffered data can be processed by the ANN. After loading the values from the RRAM memory, the memory blocks themselves can be put to sleep again during the inference of the ANN. Thereafter, the processing core can be switched off again to save energy, until enough data are buffered for the next batch. By using this technique, over 90% of the energy can be saved compared to an always-on processing core [12].

The ANN from Figure 1 uses a digital, ternary processing approach instead of mimicking the behavior of real neurons of a human brain in an analog way. The analog approach is usually realized by sending voltage pulses through a crossbar array of RRAM cells. However, while these analog processing approaches based on a crossbar structure yield promising results, the integration into a complete system can be energy-intensive [13,14]. While the calculation of one single layer with such a setup is feasible, the calculation of an entire system including input of raw data, calculation of activation functions, transfer of data to subsequent layers, and output of the classification result requires additional peripheral CMOS circuitry. Therefore, it would be necessary to convert the data from the analog to the digital domain and vice versa multiple times. This is time- and energy-intensive. When looking at a real-world applications, it is beneficial to optimize the energy consumption of the whole system rather than the individual layers. In the configuration shown in Figure 1, the conversion of the stored parameters into a digital value must be conducted only once instead of several times for each layer. Therefore, this work focuses on a digital ANN architecture with RRAM cells used as embedded, non-volatile, multi-level storage.

The presented memory block combines the RRAM cells with low-power circuitry and power gating. It offers programming operations to keep the ANN parameters flexible for possibly necessary changes of ANN parameters as well as read operations to load the saved parameters. After loading the non-volatile values from the RRAMs to latches, the RRAM block can be put to sleep. Additional design effort was invested in minimizing the leakage current during *off* cycles for lower overall power consumption. Furthermore, an operational amplifier with two output stages was designed, suited for either reading or writing, in order not to waste power for an oversized output stage during read operations while still keeping the option for programming the RRAM cells. Parallel read and write operations are possible to shorten the *on* cycle for loading the parameters, so the memory block can be put to sleep faster without wasting power. The energy consumption and all functionalities of the memory block were verified by simulation.

The article is organized as follows: In Section 2, the overall concept of the memory block is presented, and its functionality in context with ANNs is explained. In Section 3, the components of the memory block are introduced, including the RRAM memory technology and the operational amplifier. Section 4 presents the simulation results to verify the functionality of the circuit. Section 5 discusses the results and offers possibilities for adaption to other use cases, and Section 6 gives a short conclusion.

## 2. Concept Overview

**Figure 2:**
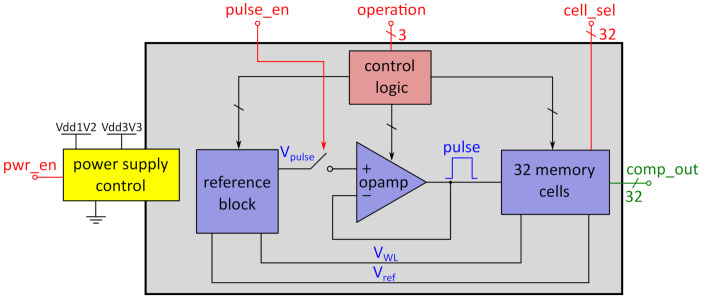
Block diagram of the memory block. The digital inputs are marked in red, the outputs in green and the internal analog signals in blue. A logic block sets several control signals for the operational amplifier (opamp), the memory cells, and the reference block. The power supply control connects or disconnects the whole memory block from the supply rails according to the power enable signal *pwr_en*.

Figure 2 shows a block diagram of the presented memory block. The 32 RRAM cells are each integrated in a memory cell, which includes the peripheral circuit for reading from the cells and is introduced in Section 3.1. The interactions with each individual cell are done via voltage pulses of specific length and height. For the design of the presented memory block, the RRAM technology of IHP–Leibniz Institut fuer innovative Mikroelektronik (IHP) was used [15,16]. The specific values for interaction with the cells are shown in Table 1. The technology uses a 1Transistor-1Resistor (1T1R) configuration, which means that the Metal–Insulator–Metal (MIM)-stack, where the resistive switching takes place, is connected in series with a transistor, that acts as a selection device as well as providing current compliance during programming via tuning the gate voltage of the transistor. Therefore, a 1T1R cell has three terminals: a Bitline (BL) terminal, which is connected to the top electrode of the MIM-stack; a Sourceline (SL), which is connected to the source terminal of the selection transistor; and the Wordline (WL), where the gate voltage of the selection transistor is applied.

For the concept of the embedded memory block, a ternary memory cell was considered to store ternary weights for the ANN application [17,18], more specifically one highly resistive state (HRS) and two low-resistive states (LRS1 and LRS2), but it is not limited to just three states and can be expanded to further multi-level operation.

The ternary application results in four different operations:

- set LRS1: program the cell from HRS to LRS1;
- set LRS2: program the cell from HRS to LRS2;
- reset: program the cell from each low resistant state (LRS1 and LRS2) to HRS;
- read: read the state of the cell without changing it.

Switching directly between LRS1 and LRS2 is not intended in this technology due to stability reasons [19]. For each of these operations, the necessary voltage levels for BL, SL, and WL are shown in Table 1. These voltage levels are provided by the reference block, shown in Figure 2. Although programming algorithms instead of single-pulse programming are common for such a memory technology, single pulses are also possible at the expense of programming precision and higher variability [20]. Since writing to the cells must only be done during retraining of the ANN, it is not crucial for timing and energy consumption during data processing. Nevertheless, is has to be stated that, in order to reduce variability, the implementation of a programming algorithm demands more voltage levels to be provided by the reference and more control circuitry. This needs to be implemented if the variability exceeds the resolution of the read operation, introduced in Section 3.

The voltage that is applied to the opamp for buffering as well as to the WL terminals of the 1T1R cells is determined by the control logic according to the operation control bits, which are applied from outside. The length of the voltage pulse is determined by the *pulse_en* signal. The buffered pulse is applied to all memory cells, which are selected by the *cell_sel* bits, which means that parallel as well as single-cell reading and programming is possible. The voltage V_ref_ is used during read operations to distinguish between the different cell states. The read operation will further be explained in Section 3.1. The read values of the comparators inside the memory cells represent the output values of the memory block and are applied to the *comp_out* terminals. Via the power supply block, the connections to the supply rails of the memory block are controlled to implement the possibility of switching off the whole circuit after reading the saved values. These values can for example be stored in a CMOS latch during operation. This enables the possibility to switch off the circuit and therefore save energy, while still maintaining the non-volatility of the stored weights as well as the possibility to write to the memory cells to grant a high degree of flexibility.

**Figure 3:**
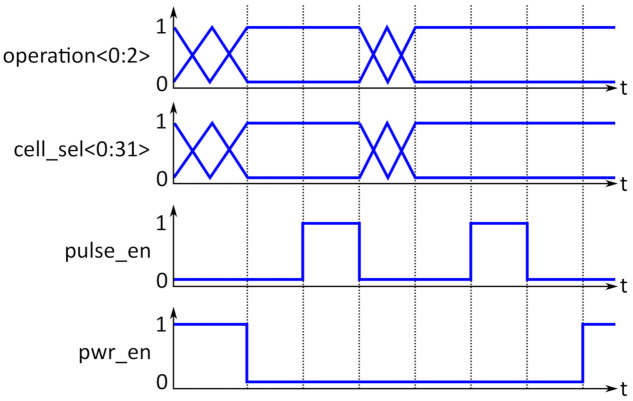
System waveforms: the *operation<0:2>* bits determine the voltage levels, while the *cell_sel<0:31>* signals activate the cells to interact with. The *pulse_en* signal triggers the operation, while *pwr_en* is used to switch the memory block on and off.

Figure 3 shows an example of waveforms for the control signals of the memory block. During this example sequence, two interactions with the RRAM cells are realized. The horizontal lines mark the clock cycles, the clock period is flexible since the system itself does not use a clock signal; therefore no specific time is stated. The specific timing and speed restrictions will be discussed in Section 4. The three operation bits determine the type of interaction: reading or programming, as well as the type of programming operation, since they demand different voltage levels (see Table 1). The *cell_sel* bits determine the cell, to which the voltage pulses will be applied. All these signals, which determine the type as well as the address for the cell interaction, should be applied before the *pwr_en* signal goes from high to low. If the *pwr_en* signal is on low lever, it connects the supply rail to the memory block. Between activating the memory block and the rising edge of the *pulse_en* signal, which triggers the buffer amplifier and therefore the voltage pulse for the cell interaction, some time should be left for the voltage reference block, to stabilize the analog voltage levels determined by the operation bits. This is shown here as a one-cycle difference between the falling edge of *pwr_en* and the rising edge of *pulse_en*. The high time of the *pulse_en* signal determines the pulse length for the voltage pulse. After the falling edge of the *pulse_en* signal, the operation and *cell_sel* bits can be readjusted for the next operation and the next pulse can be applied, again with the safety interval for stabilizing the voltage levels. After the second operation, the power of the memory block is switched off again by setting the *pwr_en* signal to high.

The sequence in Figure 3 is just an example; several read or programming pulses can be performed during one power cycle.

According to Table 1, except for the WL voltage during the reset process, no voltages above 1.2 V are necessary for operation. For two reasons, it is necessary for the buffer amplifier to be able to drive voltages above 1.2 V:

- On chip, the RRAM cells of the used technology, as well as most other RRAM technologies, show a specific degree of variability, that which makes set and reset voltages over 1.2 V necessary [21].
- During the initial forming of the cells, voltage levels exceed 1.2 V as well [22,23].

Additionally, the memory block should be suitable for other RRAM technologies that may need set and reset voltages higher than the digital logic level. Therefore, the memory block was designed with two supply voltage levels: 1.2 V as a logic level and for read operations and 3.3 V for programming and forming operations. These two levels were the default of the process in which the memory block was designed, since it offers a digital core with small transistors and a supply voltage of 1.2 V and bigger high-voltage (HV) transistors for 3.3 V supply voltage, but the concept can be applied to other technologies and systems with two types of transistors and supply voltages.

## 3. Circuit Components

This section introduces the circuit elements included in the system shown in Figure 2. All circuits were designed using IHP’s 130 nm SG13C technology utilizing the small 1.2 V transistors with a minimum gate length of 130 nm when possible and the 3.3 V HV transistors with a minimum gate length of 330 nm when necessary due to the higher voltages during forming or programming.

### 3.1. RRAM Memory Cell

**Figure 4:**
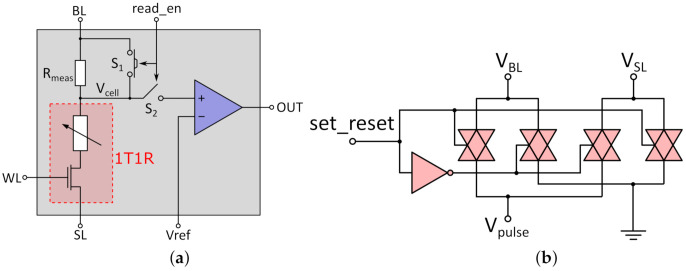
Components of the memory block: (**a**) memory cell, (**b**) set_reset switch.

Every 1T1R RRAM cell is integrated in the peripheral circuitry shown in Figure 4a. This structure will be called "memory cell" going forward, referring to the 1T1R RRAM cell as well as the circuitry around it. To understand the purpose of the circuit elements, it is useful to look at the two types of operation for the memory cell: reading and programming. The principle for reading from the cell is based on a voltage divider between the cell resistance (R_cell_) of the 1T1R cell and the measurement resistor R_meas_ in series to the cell. The read_en signal controls the two switches S_1_ and S_2_. During reading, S_1_ is open to form the voltage divider between R_cell_ and R_meas_, while S_2_ is closed, connecting the voltage between the two resistances to the comparator for evaluation. The read voltage pulse is applied to the BL terminal, while the SL is grounded. It can be assumed that no current flows into the comparator; therefore, the voltage over the cell V_cell_ (marked in Figure 4a) can be derived by:

(1) $$\frac{V_{cell}}{V_{BL}}=\frac{R_{cell}}{R_{cell}+R_{meas}}$$

The voltage V_cell_ is then compared by the comparator with V_ref_. Table 2 shows the cell resistances and the cell voltage V_cell_ during read operations for the used RRAM technology of IHP. For the measurement resistor R_meas_, a resistance of 20 kΩ was chosen. The value should lie approximately in the middle of the the expected values for R_cell_ to be distinguished to maximize the voltage differences between V_cell_ for the different states. This lowers the needed resolution for the comparator.

The necessary voltages V_ref_ to distinguish between the three states can be derived from the voltages stated in Table 2. Since multiple states must be distinguished, several read pulses are necessary to determine the cell state. As an example, at first, it could be distinguished between HRS and both LRS by setting V_ref_ between the two corresponding cell voltages, e.g., to 380 mV. A comparator output of 1 would then determine HRS, if 0 is present, a second read pulse with V_ref_ of, e.g., 250 mV distinguishes between LRS1 or LRS2. The voltage levels for V_ref_ are provided by the reference block and are applied by the control logic depending on the operation bits. This method is suitable not only for three states but for multiple states, since the reference voltage V_ref_ can be adjusted to the corresponding voltage drop over the RRAM cell. The limitation of this method is the resolution of the comparator, since it still has to evaluate the difference between the voltage drop and V_ref_, as well as the length of the read sequence, since more states have to be distinguished.

Due to a comparator in every memory cell, it is possible to read from every cell in the memory block simultaneously. This is a trade-off between longer on-time and more chip area, since the read sequence could also be done one cell at a time, sharing the comparator; however, this would lead to more energy consumption overall, since the static power consumption is dominant for a standard CMOS two-stage open-loop comparator. In general, however, both system setups are possible. Another possible read method that demands a different configuration for multi-level read operations is discussed in Section 5.

During a write operation, the read_en signal is low, closing switch S_1_ and opening S_2_ in Figure 4b. This disconnects the comparator from the RRAM cell and bypasses the measurement resistor R_meas_ to prevent any write disturbance. The applied voltage pulses and WL voltages for programming the respective states are shown in Table 1. In order to reset the cells from each LRS to HRS, the voltage pulse has to be applied to the SL while the BL is grounded in order to realize current flow through the MIM stack in the opposite direction. This will rupture the conductive filament inside the MIM stack, therefore increasing the cell resistance. The polarity of the pulse is determined by the set_reset signal, which controls the set_reset switch shown in Figure 4. The set_reset switch consists of four transmission gates that connect the BL or SL to ground and the other is connected through to the V_pulse_ terminal. The V_BL_ and V_SL_ terminals are connected to the respective terminals of the memory cell, and the voltage pulse is applied to the V_pulse_ terminal. Therefore, the bidirectional current flow for the reset process can be realized without the need to provide negative voltages on-chip and can be controlled by one single-digital signal. During read operations, the control signal should just be on set, since pulse polarity for set and read processes is identical.

In summary, the memory cell enables read and write sequences for the memory block. Since every memory cell is equipped with a comparator, parallel read operations are possible. The read sequence for multi-level cells is based on a voltage divider and comparison with a specific reference voltage and can be adjusted to more states. Due to the combination with the set_reset switch, set and reset processes can be performed without the need for negative voltages.

### 3.2. Operational Amplifier

The buffer amplifier (called “opamp” in Figure 2) used in the memory block has to drive the voltage pulses for every interaction with the memory cells. Therefore, the requirements vary depending on the operation: for reading from a single cell in HRS, it has to buffer a 500 mV pulse for a load over 100 kΩ, but when writing many cells in LRS in parallel, the load resistance can be as low as a few hundred ohms and the amplifier has to deliver a high output current. Therefor, the buffer amplifier was designed with two different output stages:

- A 1.2 V output stage to perform read operations;
- A 3.3 V output stage for programming operations.

**Figure 5:**
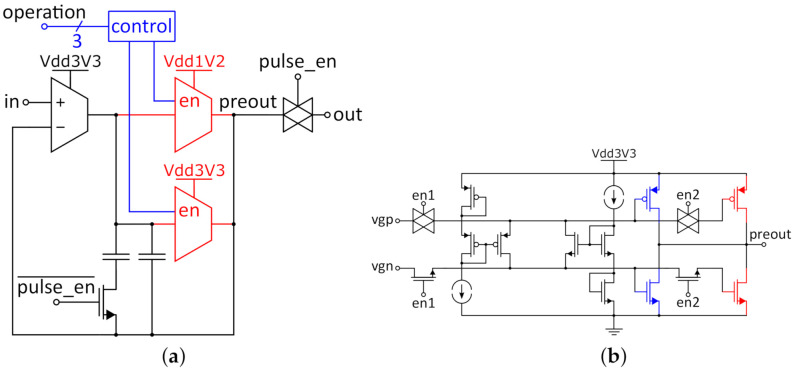
Operational Amplifier: (**a**) block diagram of the amplifier (**b**) circuit of the 3.3 V output stage.

Two output stages are activated according to the digital control signals (see Figure 5a). This has the benefit of saving energy during the read operations, since the high voltage drop over the large output transistors of the 3.3 V stage would waste a lot of energy, while the buffer amp is still able to deliver high currents during programming operations.

The input stage is designed with rail-to-rail input capability due to complementary PMOS and NMOS input transistors. The summing stage is carried out in a folded cascode configuration.

Both output stages are realized as Monticelli output stages [24]. For the 1.2 V output stage, special design effort has to be spent for the PMOS output transistor. Since the output stage is connected to the 3.3 V output stage by the preout connection, it is necessary to switch the n-well potential to 3.3 V if the output voltage exceeds 1.2 V to prevent latch up. To circumvent the body effect, which would shift the threshold voltage of the output transistor, it is not constantly connected to 3.3 V.

The 3.3 V output stage is shown in Figure 5b. The voltages “vgp” and “vgn” are provided by the input stage. The ”en1” signal connects the output stage to the input signal. In order to enable a fast wake-up time after a power-down cycle, the output transistors are divided in two parts: a small pair of output transistors (marked blue in Figure 5b) are active when the power is enabled (pwr_en = 0) while the bigger output transistors (red in Figure 5b) are just connected when a pulse must be buffered (pulse_en = 1). By that distinction, the output stage is already in the correct operating point when power is provided, without wasting a big quiescent current through the big output transistors when no pulse must be buffered, thereby saving energy. In order to keep the amplifier stable, the compensation capacitor shown in Figure 5a is connected when the big output transistors are off, since this decreases the load significantly for the first stage. When the big output transistors are active, the compensation capacity is disconnected to preserve the speed of the amplifier.

The distinction between the preout stage and the actual output of the amplifier is necessary in order to prevent undesired output voltages, since the small transistors, which are used to keep the operating point in the 3.3 V output stage, are connected to the preout as well and are active when pulse_en is deactivated.

### 3.3. Power Gating Implementation

**Figure 6:**
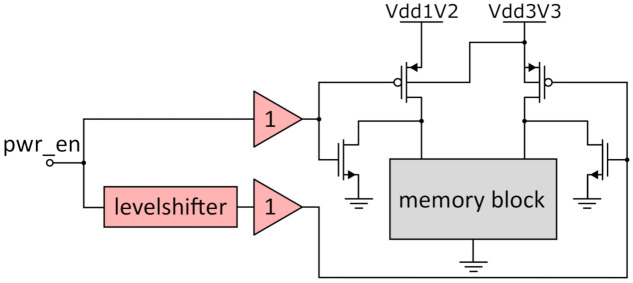
Implementation of the power control to reduce leakage current.

In order to decrease the overall power consumption significantly, it is implemented such that the memory block can be disconnected from the voltage supply. This is controlled by the *pwr_en* signal. This signal is low-active and connects or disconnects the circuit blocks from the voltage supply lines by switching off PMOS pass transistors between the circuit and the supply rails. Figure 6 shows the concept of the power control. The memory block is always connected to ground. If *pwr_en* is low, the memory block is disconnected from the supply rails but connected to the ground in order to define the voltages in the memory block during power-down cycles and prevent floating potentials. The *pwr_en* signal must be shifted to switch the 3.3 V transistor and is buffered to ensure a defined signal strength. To reduce the leakage current during power-off, the n-well of the 1.2 V transistor is connected to 3.3 V. This results in an increased body effect that raises the threshold voltage, therefore reducing the leakage current during off cycles. The channel length of the pass transistors should not be the minimal length in order to reduce the leakage current even further. To prevent a significant voltage drop over the transistors, which would lower the supply voltage, when high current is flowing into the memory block, the pass transistors should be designed with wide channel width.

## 4. Simulation Results

In the following section, the simulation results of first the operational amplifier and then the whole memory block are presented. All simulations were done using the spectre-based Virtuoso Analog Design Environment (ADE). For all simulations including RRAM cells, a model based on [25], which is adapted to the IHP RRAM technology, was used to verify correct interaction with the used technology.

### 4.1. Amplifier Simulation

**Figure 7:**
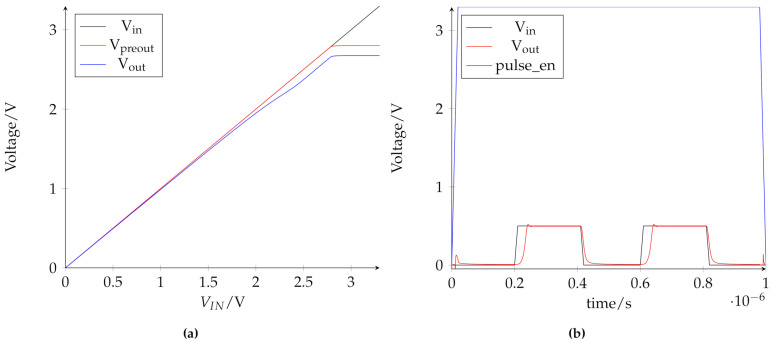
Simulation results of operational amplifier: (**a**): DC sweep of input voltage; (**b**): transient simulation of read sequence.

Figure 7a shows a DC sweep of the input voltage for the operational amplifier. The input voltage V_in_, shown in black, is applied to the positive input of the amplifier, while the negative input is connected to the output. Therefore, the amplifier is in voltage-follower configuration and always wants to replicate the input voltage at its output. The labeling of the voltages follows the description in Figure 5a. During this simulation, the 3.3V output stage is active. The load of the amplifier at the output for this simulation is a resistance of 350 Ω. This value was chosen as a worst-case estimation if all 32 RRAM cells of the memory block are selected and in LRS2. The value is calculated according to Table 2 with 32 times 13.2 kΩ in parallel and lowered by 15% to account for cell variance.

The voltage of the preout V_preout_ can reach up to 2.8 V, while the output stage can deliver up to 7.6 mA to the load. The difference between V_preout_ and V_out_ is due to the voltage drop over the transmission gate at the output of the amplifier and reaches about 130 mV at high output voltages and maximum output current for the worst-case load used in this simulation. Figure 7b shows a transient simulation of a read process performed by the amplifier. This comprises a *power_en* pulse, which activates the amplifier, followed by two voltage pulses of 500 mV. As a load for this simulation, 700 Ω were used. This is a worst-case approximation for all cells in LRS2 in parallel during a read process. The value is higher in this case, since, during read operations, the measurement resistor R_meas_, shown in Figure 4a, is always in series to the 1T1R cell. During these operations, the 1.2 V output stage is active.

The rising flank of the *pulse_en* signal activates the amplifier, which was previously in power down-mode. The two read pulses can be buffered for this worst-case load with a pulse delay between input and output of 30 ns. This determines the minimum pulse length for interaction with the memory block. Therefore, for reading, a system clock frequency of 20 MHz is possible, since this leaves enough time to apply the read pulse and do the evaluation of the voltage divider. The 1.2 V output stage delivers about 710 µA to the load, which corresponds to the applied read voltage of 500 mV multiplied with the load of 700 Ω. During a read pulse, the overall current consumption of the amplifier is 885 µA, which results in a power efficiency of 80%.

### 4.2. System Simulation

**Figure 8:**
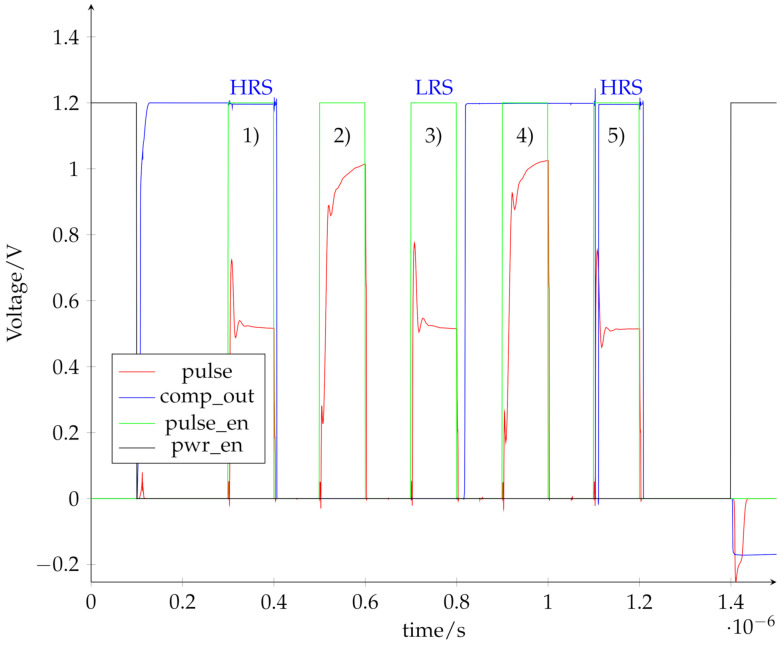
System simulation of a set and reset operation with read operations in between to evaluate the cell state.

Figure 8 shows the transient simulation results for a programming and read sequence with the whole memory block. The nomenclature of the signals is taken from Figure 2. The *pwr_en* signal (yellow) connects the memory block to the voltage supply. The *pulse_en* signals (green) marks the voltage pulses while the *pulse* signal (red) shows the voltage at the output of the amplifier, which is directly applied to the RRAM cells. As a pulse width, 100 ns were chosen. The simulation sequence contains the following operations, marked in Figure 8:

(1) reading (0.3 µs– 0.4 µs)
(2) set LRS2 (0.5 µs– 0.6 µs)
(3) reading (0.7 µs– 0.8 µs)
(4) reset (0.9 µs– 1 µs)
(5) reading (1.1 µs– 1.2 µs)

The *comp_out* signal (blue) is used to read the cell status and determines, in this case, if the cell is in either HRS, if *comp_out* is on high level, or one of the LRS, if *comp_out* is on low level. The signal is not valid during a set or reset operation.

The first read operation (1) determines that the cell is initially in HRS (*comp_out* = 1). This is followed by a set operation (2) that programs the selected cell to LRS2, which is verified by the following read operation (3), where *comp_out* = 0 determines the LRS. After the second read operation, a reset (4) is applied, to program HRS, which is again verified by the third read operation (5) (*comp_out* = 1). The simulation shows that the system can successfully read and program the RRAM cells.

The pulse voltage in Figure 8 needs significant settling time, which becomes more obvious during the programming operations. This is caused by the voltage reference, which is composed of a string of matching resistors. Therefore, if there are switches in the tapped voltage, the reference needs time to resettle again. If a higher bandwidth reference circuit is implemented, the settling time can be reduced. Higher bandwidth usually means more current consumption, so there is a trade off between power consumption and settling time of the voltage pulses. Since the system is able to program the cells, the low bandwidth of the reference circuit is sufficient in this case.

In terms of energy efficiency, as explained in Section 3.3, power gating for the memory block was implemented. During power down, which is controlled by the *pwr_en* signal, the whole memory block leaks 1.086 nA overall, which is separated in 774 pA leakage from the 1.2 V supply rail and 312 pA leakage from the 3.3 V supply rail. This results in a overall power consumption due to leakage of 1.95 nW, when the whole memory block is in stand-by mode.

Another important metric of the memory block is the energy needed for one read sequence. As established in Section 3.1, reading was sequenced in two read operations to determine first HRS or LRS and then LRS1 or LRS2. The operation shown in Figure 7b depicts such a sequence. The energy needed for one read sequence is 1.127 nJ. This includes the parallel reading of all 32 cells in parallel and take into account all needed operations from switching on the memory block until stand-by mode.

## 5. Discussion

The presented RRAM block was designed for and simulated with a specific RRAM technology as well as the presented application as weight storage in ANNs as primary focus. Nevertheless, there are several degrees of freedom, that should be considered during the design process, which can be adapted while still using the same overall concept. The following are the main discussion points that can be considered:

- serial or parallel operations
- multi-level capability
- number of cells in one memory block
- allocation of reference voltage levels

In the following paragraphs, these points will briefly be discussed to show some design variability for the presented memory concept.

### 5.1. Serial or Parallel Operations

The presented memory block has the capability to read or program all cells in parallel, with the restriction that only the same operation can be performed simultaneously. For example, all selected cells can just be programmed to LRS1 in parallel, but not one to LRS1 and another cell to LRS2 at the same time. If simultaneous operations are not necessary, the memory block can save significant circuitry:

- The comparators in each memory cell can be reduced to one single comparator, since no parallel read operations are necessary.
- Due to the significantly reduced variety of load conditions for the operational amplifier, which is needed to drive the resistive loads, the requirements for the amplifier are reduced. This can enable a design with lower power consumption, mainly due to the smaller output stage, since lower currents need to be provided during single cell operations.

The trade-off between serial and parallel operations is between longer on-time and less power consumption during on-time. The optimum of this trade-off is dependent on the specific design of the circuit elements as well as overall system design and process characteristics and can be subject of future research.

### 5.2. Multi-Level Capability

If the memory cells can hold more than two states, there are two ways to distinguish the different states during read operations using the method described in Section 3.1, which discriminates according to the voltage drop over a measurement resistor:

1. A sequence of several read operations that compares the voltage drop with different reference voltages. This method was used in the presented memory block.
2. Introducing more comparators per memory cell in order to compare the voltage drop over the measurement resistor simultaneously to several voltages to determine the cell state in one single read operation.

For the first method, the circuit effort is lower since only one comparator is needed. Additionally, the information of the first comparison can be used in the next read sequence to apply a comparison voltage accordingly. Therefore, the number of distinguishable states depending on number of read operations can be calculated as:

(2) $$N_{states}=2^{N_{operations}}$$

where $N_{states}$ is the amount of distinguishable states and $N_{operations}$ is the number of needed read operations.

For the second method, it is not possible to use the information of the former read steps; therefore the number of states depending on the needed comparators is:

(3) $$N_{states}=N_{comparators}+1,$$

where $N_{states}$ is the number of distinguishable states and $N_{comparators}$ is the number of needed comparators or simultaneous compare operations. The specific energy comparison is again dependent on the circuit design of the compare operation, but in general, from a circuit perspective, sequenced read operations to determine multi-level states can save energy and chip area due to the lower number of circuit elements needed. This effect becomes more significant with a higher number of possible states per cell. On the other hand, if timing is crucial or chip area not an issue, the read operation for multi-level RRAM cells can be parallelized at the price of higher hardware cost.

### 5.3. Number of Cells per Memory Block

The number of 1T1R cells in one memory block can be altered as well. This has implications on the requirements for the amplifier as well as consequences for area consumption and routing effort. The amplifier requirements are strongly influenced by the method of reading and programming discussed above with respect to the block size: if no parallel operations need to be implemented, the specification for the amplifier is basically independent of the block size (except for the slightly higher parasitics due to generally longer routing lines), while the block size has a very high impact for parallel operations, since the load for the amplifier can then vary over a wider range with increased number of parallel cells. Since more cells need to be connected, the routing effort increases linearly with higher number of cells per block. However, with a higher number of cells, the overall number of memory blocks can be decreased, which lowers the requirements for the control logic, which selects and determines the operation of the different memory blocks in the whole system. This is highly dependent on the application and should be evaluated with respect to the area of operation.

### 5.4. Allocation of Reference Voltage Levels

The allocations of the needed voltage levels for the different operations can be organized per block (as chosen in the presented memory block) or as one central reference for the whole system. A central reference has higher requirements since the output voltages have to be distributed over the whole system to each individual memory block, which results in a greater electrical load for the reference as well as more routing effort for the overall system. Additionally, the interface for the individual memory block also contains the analog voltage levels; therefore, it is not purely digital and the system has to deal with analog signals on the top level. As a benefit, the quality of the central voltage reference can be improved without investing the energy cost in every memory block but only once in the central reference circuit. A central reference furthermore decreases the area consumption of the individual blocks. As a rough estimate, with a smaller number of memory blocks in the system, the advantages of a central reference can be more easily taken advantage of due to decreased routing effort and higher reference quality, while with a higher and more distributed number of blocks, the routing effort and load requirements are increased.

## 6. Conclusions

This paper presents a low-power RRAM memory block suited for distributed, embedded weight, and bias storage for ANNs. The presented memory block provides both read and programming ability as well as multi-level capability. Due to parallel operation and an implemented power gating ability, the memory block can provide the stored information with low power consumption. In power-down mode, the memory block has a leakage power of 1.95 nW. One multi-level read operation consumes 1.127 nJ while providing all information of the memory block. Due to the non-volatility of the RRAM cells, the information is also preserved with unstable power supply.

Due to the structure of the memory block, this concept can provide a high degree of versatility. The operational amplifier enables flexibility for different read, programming, and forming algorithms, while the voltage reference can provide different voltage levels if necessary. Based on this versatility provided by the peripheral circuitry, this concept can be easily adapted to different applications or RRAM technologies.

## Figures and Tables

**Table 1:** Voltage levels for cell operations.

Operation	V_BL_	V_SL_	V_WL_
read	0.5 V	0 V	1.2 V
set LRS1	1 V	0 V	0.8 V
set LRS2	1 V	0 V	1.2 V
reset	0 V	1 V	2.7 V

**Table 2:** RRAM cell state resistances and read voltages.

State	R_cell_	V_cell_
HRS	196 kΩ	453 mV
LRS1	33.7 k Ω	310 mV
LRS2	13.2 k Ω	196 mV

## Data Availability

Simulation and measurement data is available on request from the corresponding author.

## Abbreviations

The following abbreviations are used in this manuscript:

ANN	Artificial Neural Network
AFib	Atrial Fibrillation
ECG	Electrocardiogram
RRAM	Resistive Random Access Memory
1T1R	One Transistor/One Resistor
MIM	Metal-Insulator-Metal
BL	Bitline
SL	Sourceline
WL	Wordline
HRS	High Resistive State
LRS	Low Resistive State
